# Intravoxel Incoherent Motion Magnetic Resonance Imaging Used in Preoperative Screening of High-Risk Patients With Moyamoya Disease Who May Develop Postoperative Cerebral Hyperperfusion Syndrome

**DOI:** 10.3389/fnins.2022.826021

**Published:** 2022-03-02

**Authors:** Feng Gao, Wei Zhao, Yu Zheng, Yu Duan, Ming Ji, Guangwu Lin, Zhenfang Zhu

**Affiliations:** ^1^Department of Radiology, Huadong Hospital Fudan University, Shanghai, China; ^2^Department of Radiology, The Second Xiangya Hospital of Central South University, Changsha, China; ^3^Department of Radiology, Chengdu Second People’s Hospital, Chengdu, China; ^4^Department of Neurosurgery, Huadong Hospital Fudan University, Shanghai, China

**Keywords:** moyamoya disease, perfusion imaging, magnetic resonance imaging, microvascular circulation, surgery

## Abstract

**Objective:**

This study aimed to investigate the feasibility of preoperative intravoxel incoherent motion (IVIM) MRI for the screening of high-risk patients with moyamoya disease (MMD) who may develop postoperative cerebral hyperperfusion syndrome (CHS).

**Methods:**

This study composed of two parts. In the first part 24 MMD patients and 24 control volunteers were enrolled. IVIM-MRI was performed. The relative pseudo-diffusion coefficient, perfusion fraction, apparent diffusion coefficient, and diffusion coefficient (rD*, rf, rADC, and rD) values of the IVIM sequence were compared according to hemispheres between MMD patient and healthy control groups. In the second part, 98 adult patients (124 operated hemispheres) with MMD who underwent surgery were included. Preoperative IVIM-MRI was performed. The rD*, rf, rADC, rD, and rfD* values of the IVIM sequence were calculated and analyzed. Operated hemispheres were divided into CHS and non-CHS groups. Patients’ age, sex, Matsushima type, Suzuki stage, and IVIM-MRI examination results were compared between CHS and non-CHS groups.

**Results:**

Only the rf value was significantly higher in the healthy control group than in the MMD group (*P* < 0.05). Out of 124 operated hemispheres, 27 were assigned to the CHS group. Patients with clinical presentation of Matsushima types I–V were more likely to develop CHS after surgery (*P* < 0.05). The rf values of the ipsilateral hemisphere were significantly higher in the CHS group than in the non-CHS group (*P* < 0.05). The rfD* values of the ACA and MCA supply areas of the ipsilateral hemisphere were significantly higher in the CHS group than in the non-CHS group (*P* < 0.05). Only the rf value of the anterior cerebral artery supply area in the contralateral hemisphere was higher in the CHS group than in the non-CHS group (*P* < 0.05). The rf values of the middle and posterior cerebral artery supply areas and the rD, rD*, and rADC values of the both hemispheres were not significantly different between the CHS and non-CHS groups (*P* > 0.05).

**Conclusion:**

Preoperative non-invasive IVIM-MRI analysis, particularly the *f*-value of the ipsilateral hemisphere, may be helpful in predicting CHS in adult patients with MMD after surgery. MMD patients with ischemic onset symptoms are more likely to develop CHS after surgery.

## Introduction

Moyamoya disease (MMD) is a common neurological disease characterized by progressive steno-occlusion of the distal internal carotid artery, leading to the compensatory development of collateral vessels (moyamoya vessels) at the base of the brain (Suzuki and Takaku, 1969). MMD is more prevalent in the Asian population and more frequently occurs in women. Revascularization is recommended for patients with MMD (Research Committee on the Pathology and Treatment of Spontaneous Occlusion of the Circle of Willis, and Health Labour Sciences Research Grant for Research on Measures for Infractable Diseases, 2012). Superficial temporal artery-middle cerebral artery (STA-MCA) bypass in combination with indirect procedures has been employed as treatment for patients with MMD (Kazumata et al., 2014). Although an STA-MCA bypass carries a relatively small amount of blood, cerebral hyperperfusion is frequently observed after revascularization surgery, particularly in adult patients (Zhao et al., 2013; Kazumata et al., 2014). Furthermore, patients with MMD are more likely to develop cerebral hyperperfusion syndrome (CHS) after revascularization surgery than those with other atherosclerotic occlusive cerebrovascular diseases (Fujimura et al., 2011). Postoperative complications in patients with MMD can cause not only postoperative neurological symptoms but also permanent neurological defects (Cho et al., 2014; Antonucci et al., 2016; Kim et al., 2016). Postoperative CHS in MMD can lead to an increase in intracranial vascular pressure and may induce intracranial hemorrhage and even hematogenous cerebral edema (Mesiwala et al., 2008; Park et al., 2018). Studies have shown that appropriately lowering the patients’ blood pressure during the perioperative period may reduce the incidence of postoperative CHS (Hayashi et al., 2012). In clinical practice, lowering the blood pressure of patients with MMD after surgery may also lead to cerebral vascular insufficiency and cerebral infarction (Lee et al., 2018). Identifying patients with MMD who are at high risk for CHS development after surgery and implementing appropriate perioperative management are important to prevent the occurrence of postoperative hyperperfusion and improve the prognosis of these patients (Ostergaard et al., 2014).

Impaired cerebral autoregulation has been implicated in the mechanism of cerebral hyperperfusion mediated by endothelial dysfunction. Various imaging modalities such as single-photon emission computed tomography (SPECT), computed tomography (CT), and magnetic resonance imaging (MRI) can determine the cerebral perfusion status. SPECT and CT perfusion are radiative, whereas MRI techniques, including dynamic contrast-enhanced MRI, dynamic susceptibility contrast-enhanced (DSC) MRI, arterial spin labeling (ASL) MRI, and methods used to analyze cerebral microcirculation, such as intravoxel incoherent motion (IVIM) MRI, and vascular space occupancy are non-radiative (Callewaert et al., 2021). Brain IVIM technique is advantageous over other technique, such as positron emission tomography, CT, DSC MRI, and ASL MRI, because it does not require contrast media injection or radiation exposure and is not affected by the arterial input (Hara et al., 2019). Microvessels are below the currently achievable spatial resolution of MRI and other clinically applicable imaging methods, making the direct non-invasive measurement of microvascular density impossible to be performed. Nonetheless, MRI can be applied to measure microvascular perfusion, providing indirect readouts of the underlying microvasculature alteration (Federau et al., 2012; Callewaert et al., 2021).

IVIM sequence, which was proposed by Le Bihan et al. (1986), is a diffusion MRI technique that can simultaneously provide information regarding both molecular diffusion and perfusion (Le Bihan, 2019). Microscopic movement in organisms includes the diffusion of water molecules and microcirculation of blood. IVIM is a double exponential model that divides biological tissues into two components: a slow-moving component in which water molecules diffuse based on Brownian motion and a fast-moving component in which water molecules move due to blood circulation (Federau et al., 2012). Four parameters, including apparent diffusion coefficient (ADC), diffusion coefficient (D), pseudo-diffusion coefficient (D*), and perfusion fraction (f), can be can be obtained from IVIM sequence. Promising results had been obtained when perfusion was measured using the IVIM technique in various diseases of different body organs, such as liver (Guiu et al., 2012), kidney (Thoeny et al., 2009), pancreas (Lemke et al., 2009), breast (Sigmund et al., 2011), prostate (Riches et al., 2009), and head and neck tumors (Sumi and Nakamura, 2013). The application of IVIM technique to the brain has increased over recent years (Paschoal et al., 2018). Previous study showed that f and fD* are more sensitive to detect changes during the stroke process, as these parameters are more related to cerebral blood flow (CBF) (Yao et al., 2016). IVIM also can be used to assessment of patients with cerebral small vessel disease (Wardlaw et al., 2013). Hara et al. (2019) used IVIM to evaluate the hemodynamic disturbance of MMD by comparison with the gold-standard ^15^O-gas positron emission tomography method. All those studies showed that IVIM may be used to non-invasively assess cerebral hemodynamic impairment in patients with MMD. To the best of our knowledge, thus far, no study has used preoperative IVIM-MRI to predict patients with MMD who may develop postoperative CHS. This study aimed to investigate potential risk factors for CHS after surgery in patients with MMD using the IVIM technique.

## Materials and Methods

### Clinical Data and Participants

This study was approved by the ethics committee of our hospital. Written informed consent was obtained from all patients. All experiments were performed in accordance with the relevant guidelines and regulations set by the ethics committee. This study was composed of two parts. First part compared parameters of MMD patient and healthy control groups. Second part only included MMD patients, preoperative parameters between CHS and non-CHS group were compared.

From January 2018 to July 2018, 24 adult patients with MMD (mean age, 41.67 ± 10.606; range, 19–65 years; female, 12; male, 12) with 48 brain hemispheres and 24 healthy adult volunteers (mean age, 43.67 ± 8.499; range, 19–65 years; female, 14; male, 10) with 48 brain hemispheres were included in this study. MMD was diagnosed using digital substraction angiography (DSA) according to the criteria of the Research Committee on Spontaneous Occlusion of the Circle of Willis (MMD) of the Ministry of Health and Welfare, Japan (Fukui, 1997). The inclusion criteria for the MMD group were as follows: (i) patients aged > 18 years and (ii) patients with no contraindications to MR examination. All healthy volunteers were from our hospital and underwent routine physical examinations. The inclusion criteria for the healthy control group were as follows: (i) patients aged > 18 years; (ii) no previous neurological history; (iii) MRI examination revealing no obvious hemorrhagic foci or encephalomalacia; (iv) no autoimmune disease; (v) magnetic resonance angiography examination showing no obvious stenosis and occlusion of cerebral arteries; and (vi) patients with no contraindications to MRI.

From August 2018 to May 2021, 98 adult patients with MMD (mean age, 42.87 ± 11.32 years; range, 19–65 years; female, 52; male, 46) with 124 operated hemispheres were included in this study (26 patients underwent bilateral revascularization surgeries, 72 patients underwent unilateral revascularization surgeries. Patients with bilateral cerebral hemispheres that both meet surgical indications, the side with more severe clinical symptoms or more worse perfusion status will be operated first.). All MR examinations were performed within 1 week before the revascularization surgery. MMD was diagnosed using DSA (Fukui, 1997). The inclusion criteria were as follows: (i) patients aged > 18 years; (ii) patients with no contraindications to MR examination; and (iii) patients who underwent STA-MCA bypass and encephalo-duro-myo-synangiosis (EDMS) combined surgery. In our hospital, most patients underwent combined surgery, although only a few patients underwent EDMS surgery. Therefore, we only included patients who underwent combined surgery in this study to avoid bias caused by different surgery types.

### Magnetic Resonance Imaging Examination Protocol and Image Analysis

All preoperative MR examinations were performed with a 3-T whole-body MRI scanner (Siemens Skyra Freedom; Siemens Medical Solutions, Erlangen, Germany) using a head-neck coil. All patients were instructed to stay still and not to think about anything with their eyes closed during the MRI examination. T1-weighted imaging, T2-weighted imaging, fluid-attenuated inversion-recovery sequence, time-of-flight magnetic resonance angiography, and IVIM-MRI were performed in this study.

### Intravoxel Incoherent Motion Scanning Protocol and Imaging Analysis

**FIGURE 1:**
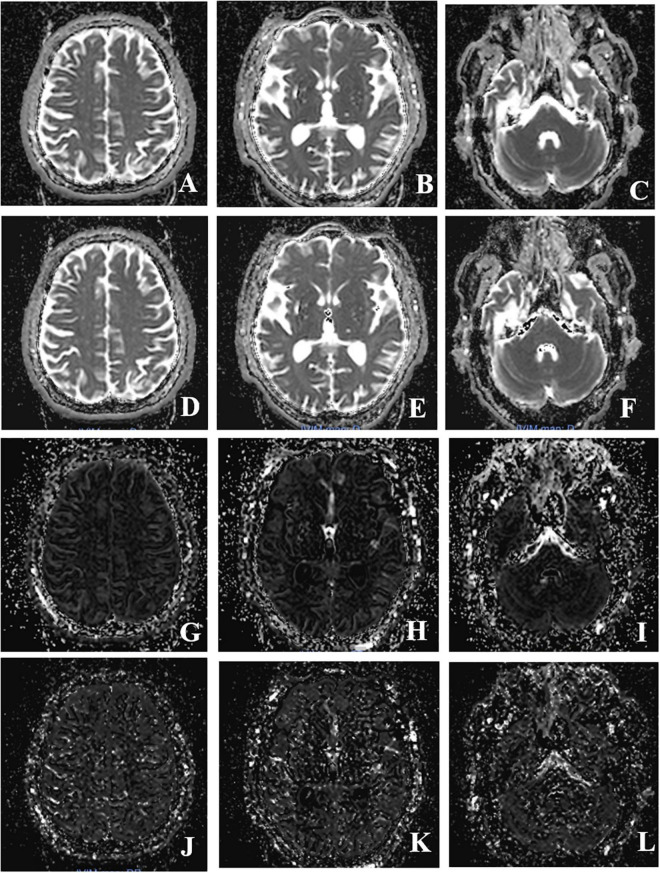
Intravoxel incoherent motion MRI imaging obtained after post-processing (the columns show three different levels, peripheral white matter level, basal ganglia level, cerebellar hemisphere level; the line shows four different IVIM parameters, ADC, D, f, D*value). **(A–C)** Image of ADC value; **(D–F)** image of *D*-value; **(G–I)** image of f value; **(J–L)** image of D* value.

**FIGURE 2:**
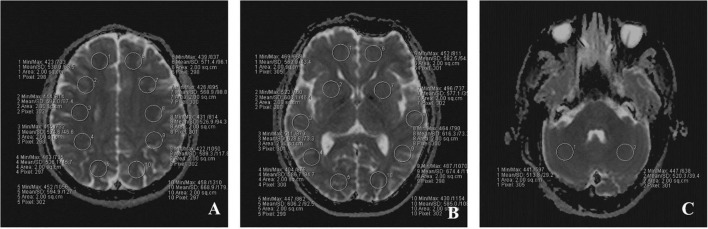
The placement of ROIs on IVIM images in three different level. **(A)** peripheral white matter level; **(B)** basal ganglia level; **(C)** cerebellar hemisphere level. A total of 22 ROIs were placed symmetrically on the three transverse images.

Multiple *b*-values were used (0, 20, 40, 80, 110, 140, 170, 200, 300, 400, 500, 600, 700, 800, 900, and 1,000 s/mm^2^) in the IVIM sequence. Detailed scanning parameters are listed in Table 1. Four parameters, including apparent diffusion coefficient (ADC), diffusion coefficient (D), pseudo-diffusion coefficient (D*), and perfusion fraction (f), were calculated and derived using a Siemens IVIM work-in-progress package (MR Body Diffusion Toolbox version 1.3.0) (Figure 1). Regions of interest (ROIs) with dimensions of approximately 200 mm^2^ were placed manually to avoid subarachnoid spaces, cerebral infarction, and cerebral hemorrhage areas. As ROIs in this study were placed manually, to ensure the accuracy of measurement, the ROIs were place three times for each patient, and the average value was considered as the final result. Each cerebral hemisphere was divided into three areas according to the different supply cerebral arteries [anterior cerebral artery (ACA) supply area, MCA supply area, and posterior cerebral artery (PCA) supply area]. A total of 22 ROIs were placed symmetrically on the peripheral white matter, basal ganglia, and cerebellar hemisphere levels (Togao et al., 2006; Zaharchuk et al., 2011). Moreover, the ipsilateral cerebellar hemisphere was used as a reference to standardize the evaluated parameters. Subsequently, rADC, rD, rD*, and rf were obtained [i.e., rADC = ADC (different blood artery supply area)/ADC (ipsilateral cerebellar hemisphere) (Figure 2)].

**TABLE 1:** MRI scanning sequences and parameters.

	TR/TE (ms)	Thickness (mm)	Flip angle (°)	Intersection gap	TA	FOV (cm^2^)	Matrix	*b*-value
T1WI	230/2.46	5	70	30%	25 s	22 × 22	256 × 192	/
T2WI	5,000/117	5	90	30%	54 s	22 × 22	384 × 281	/
FLAIR	8,000/85	5	140	20%	1 min 36 s	22 × 22	256 × 162	/
DWI	1,300/62	5	192	30%	30 s	24 × 24	192 × 192	0.1000
TOF	21/3.43	1	18	−18.75%	5 min 13 s	22 × 22	320 × 180	/
IVIM	5,100/92	5	/	30%	5 min 22 s	22 × 22	130 × 130	16 *b*-value

*TR, time of repetition; TE, time of echo; FOV, field of view; TA, time of acquisition; FLAIR, fluid-attenuated inversion-recovery sequence; DWI, diffusion-weighted imaging; IVIM, intravoxel incoherent motion. The 16 b-values are 0, 20, 40, 80, 110, 140, 170, 200, 300, 400, 500, 600, 700, 800, 900, and 1,000 s/mm^2^.*

### Diagnosis of Cerebral Hyperperfusion Syndrome and Group Analysis

In the first part, the participants were divided into an MMD patient group and a healthy control volunteer group. All parameters were compared between the two groups. The rD*, rf, rADC, and rD values of the IVIM sequence were compared according to hemisphere between the MMD and healthy control groups. The D*, f, ADC, and D values were compared according to cerebellum between the MMD and healthy control groups.

In the second part, symptomatic CHS was defined as the postoperative development of a severe headache, seizures, and new neurological deficits, with neither definite hematomas nor definite acute infarctions presenting or observed on brain CT images, diffusion magnetic resonance images, or both (van Mook et al., 2005; Iwata et al., 2014). A senior neurosurgeon blinded to the study confirmed the diagnosis of postoperative CHS. Operated hemispheres were divided into CHS and non-CHS groups based on whether the patient developed CHS after surgery. We then compared the parameters between the CHS and non-CHS groups, and each hemisphere was divided according to different supply areas: the ACA, MCA, and PCA supply areas. In this section, the rD*, rf, rADC, rD, and rfD* values of the IVIM sequence were compared according to different cerebral artery supply areas in the two hemispheres (ipsilateral/surgery side and contralateral/non-surgery side).

### Statistical Analyses

The independent *t*-test or Mann–Whitney *U*-test, where appropriate, was used to assess between-group differences in continuous variables (age as well as the rD*, rf, rADC, and rD values of the IVIM sequence). The chi-square test was applied to evaluate between-group differences in categorical variables [sex, hypertension, diabetes, Suzuki stage (Suzuki and Takaku, 1969), and Matsushima type (Matsushima et al., 1990, Supplementary Table 1). The IVIM parameters showed statistically significant differences between the CHS and non-CHS groups; the receiver operating characteristic (ROC) curve was analyzed to obtain the diagnostic efficacy of these parameters. Statistical results were considered significant when the *P*-values were < 0.05. Statistical analyses were performed using a commercially available computer software program (IBM SPSS version 22).

## Results

The rD, rD*, rADC, and rf values of the MMD and healthy control groups are shown in Table 2. Only the rf value was significantly higher in the healthy control group than in the MMD group (*P* < 0.05). No significant differences were found in rADC, rD, and rD* between the MMD and healthy control groups (*P* > 0.05). No significant differences were found in ADC, f, D, and D* of cerebellum between the MMD and healthy control groups (*P* > 0.05).

**TABLE 2:** Examination results of the MMD and control groups.

	Control	MMD		*P*
Sex (n)			χ^2^ = 0.671	0.413
Male (n)	10	12		
Female (n)	14	12		
Age (Year)	43.67 ± 8.499	41.67 ± 10.606	*t* = 1.020	0.311
**IVIM**				
rADC	1.183 ± 0.091	1.202 ± 0.142	*t* = −0.792	0.430
rf	1.345 ± 0.211	1.229 ± 0.211	*t* = 2.688	0.008
rD	1.181 ± 0.095	1.212 ± 0.124	*t* = −1.353	0.179
fD*	1.045 ± 0.173	1.080 ± 0.169	*t* = −0.984	0.328
**IVIM (cerebellum)**				
ADC (μm^2^/s)(× 10^6^)	687.396 ± 38.176	700.354 ± 55.802	*t* = −1.328	0.187
f (μm^2^/s)(× 10^3^)	58.454 ± 14.288	54.623 ± 8.566	*t* = 1.593	0.115
D (μm^2^/s)(× 10^6^)	657.654 ± 35.982	658.690 ± 25.632	*t* = −0.162	0.871
D* (μm^2^/s)(× 10^4^)	97.746 ± 15.869	103.225 ± 14.969	*t* = −1.741	0.085

ADC, apparent diffusion coefficient; D, diffusion coefficient; D*, pseudo-diffusion coefficient; f, perfusion fraction.

There were 27 (27/124, 21.77%) operated hemispheres developed CHS after revascularization surgery. The main symptoms of CHS included severe headache (six cases), persistent vomiting (two cases), dysphagia (three cases), speech impairment (seven cases), face and eye pain (one case), muscle strength decrease (the upper limbs in two cases and the lower limbs in four cases), and seizure (two cases). These symptoms were observed 1–9 days (mean ± standard deviation: 3.444 ± 2.532 days) after combined surgery. All symptoms improved before discharge from the hospital.

The CHS and non-CHS groups exhibited a significant difference with respect to the Matsushima type (*P* < 0.05). Overall, 88.89% (24/27 operated hemispheres) of operative patients in the CHS group presented with Matsushima types I–V, whereas 58.76% (57/97 operated hemispheres) of operative patients in the non-CHS group presented with Matsushima types I–V. No statistically significant differences in age, sex, hypertension, diabetes, and Suzuki stage were observed between the CHS and non-CHS groups (*P* > 0.05; Table 3).

**TABLE 3:** Clinical characteristics of the patients in the CHS and non-CHS groups.

	CHS	Non-CHS		*P*
No. of patients (%)	27 (21.77%)	97 (78.23%)		
Age (Year)	46.741 ± 11.918	42.258 ± 10.969	*t* = −1.843	0.068
Sex			χ^2^ = 0.987	0.321
Male (n)	11	50		
Female (n)	16	47		
Hypertension	13	39	χ^2^ = 0.547	0.459
Diabetes	11	32	χ^2^ = 0.560	0.454
Matsushima types			χ^2^ = 11.194	0.048
I	2	6		
II	2	10		
III	7	15		
IV	5	14		
V	8	12		
VI	3	40		
Suzuki stages			χ^2^ = 2.992	0.559
1	0	0		
2	2	4		
3	11	30		
4	7	41		
5	7	21		
6	0	1		

*CHS, cerebral hyperperfusion syndrome; non-CHS, non-cerebral hyperperfusion syndrome.*

The preoperative microvascular perfusion status of the CHS and non-CHS groups is shown in Table 4. The hemispheres were divided into ipsilateral (hemispheres performed with surgery) and contralateral hemispheres. The rf values of the ACA, MCA, and PCA supply areas of the ipsilateral hemisphere were significantly higher in the CHS group than in the non-CHS group (*P* < 0.05). Furthermore, rADC, rD, and rD* in all areas of the ipsilateral hemisphere showed no significant differences between the CHS and non-CHS groups (*P* > 0.05). Only the rf value of the ACA supply area in the contralateral hemisphere was significantly higher in the CHS group than in the non-CHS group (*P* < 0.05). The rf values of the MCA and PCA supply areas of the contralateral hemisphere were not significantly different between the CHS and non-CHS groups (*P* > 0.05). The values of rADC, rD, and rD* in all areas of the contralateral hemisphere were not significantly different between the CHS and non-CHS groups (*P* > 0.05). The rfD* values of the ACA and MCA supply areas of the ipsilateral hemisphere were significantly higher in the CHS group than in the non-CHS group (*P* < 0.05). Furthermore, rfD* value of PCA supply areas of ipsilateral hemisphere and in all areas of the contralateral hemisphere showed no significant differences between the CHS and non-CHS groups (*P* > 0.05).

**TABLE 4:** Preoperative microvascular perfusion status of the CHS and non-CHS groups.

	Non-CHS	CHS		*P*
**Ipsilateral**				
rADC-ACA	1.155 ± 0.12	1.184 ± 0.087	*t* = −1.135	0.258
rADC-MCA	1.267 ± 0.327	1.309 ± 0.163	*t* = −0.642	0.522
rADC-PCA	1.228 ± 0.160	1.293 ± 0.191	*t* = −1.794	0.075
rf-ACA	1.090 ± 0.275	1.294 ± 0.295	*t* = −3.355	0.001
rf-MCA	1.288 ± 0.296	1.551 ± 0.371	*t* = −3.863	0.000
rf-PCA	1.130 ± 0.317	1.328 ± 0.331	*t* = −2.847	0.005
rD-ACA	1.172 ± 0.124	1.181 ± 0.077	*t* = −0.355	0.723
rD-MCA	1.257 ± 0.175	1.303 ± 0.154	*t* = −1.238	0.218
rD-PCA	1.242 ± 0.154	1.292 ± 0.193	*t* = −1.398	0.165
rD*-ACA	1.064 ± 0.219	1.100 ± 0.257	*t* = −0.736	0.463
rD*-MCA	1.083 ± 0.227	1.141 ± 0.333	*t* = −1.052	0.295
rD*-PCA	0.963 ± 0.205	0.968 ± 0.185	*t* = −0.105	0.917
rfD*-ACA	1.175 ± 0.457	1.441 ± 0.556	*t* = −2.553	0.012
rfD*-MCA	1.411 ± 0.533	1.785 ± 0.732	*t* = −2.955	0.004
rfD*-PCA	1.102 ± 0.482	1.287 ± 0.422	*t* = −1.809	0.073
**Contralateral**				
rADC-ACA	1.152 ± 0.108	1.162 ± 0.097	*t* = −0.424	0.672
rADC-MCA	1.199 ± 0.126	1.224 ± 0.136	*t* = −0.917	0.361
rADC-PCA	1.228 ± 0.213	1.234 ± 0.172	*t* = −0.136	0.892
rf-ACA	1.109 ± 0.236	1.210 ± 0.212	*t* = −1.993	0.048
rf-MCA	1.278 ± 0.352	1.346 ± 0.291	*t* = −0.919	0.360
rf-PCA	1.143 ± 0.286	1.237 ± 0.251	*t* = −1.555	0.122
rD-ACA	1.162 ± 0.102	1.164 ± 0.095	*t* = −0.097	0.923
rD-MCA	1.207 ± 0.123	1.224 ± 0.130	*t* = −0.623	0.534
rD-PCA	1.251 ± 0.220	1.232 ± 0.173	*t* = 0.417	0.677
rD*-ACA	1.052 ± 0.184	1.090 ± 0.236	*t* = −0.767	0.448
rD*-MCA	1.078 ± 0.180	1.076 ± 0.205	*t* = 0.030	0.976
rD*-PCA	0.988 ± 0.207	0.958 ± 0.214	*t* = 0.662	0.509
rfD*-ACA	1.164 ± 0.309	1.331 ± 0.426	*t* = −1.902	0.066
rfD*-MCA	1.385 ± 0.486	1.465 ± 0.466	*t* = −0.768	0.444
rfD*-PCA	1.127 ± 0.354	1.202 ± 0.430	*t* = −0.931	0.354

*ACA, anterior cerebral artery supply area; MCA, middle cerebral artery supply area; PCA, posterior cerebral artery supply area.*

**FIGURE 3:**
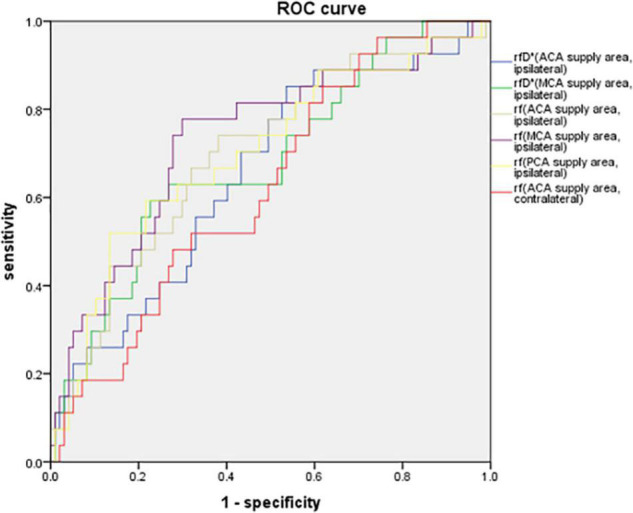
ROC curve of rfD* in the ACA, MCA supply areas of the ipsilateral hemisphere, rf in the ACA, MCA, PCA supply areas of the ipsilateral hemisphere and in the ACA supply area of the contralateral hemisphere for predicting postoperative CHS. The AUCs of rf in the ACA, MCA, and PCA supply areas of the ipsilateral hemisphere for predicting postoperative CHS were 0.700, 0.733, and 0.701, respectively. The AUC for rf in the ACA supply area in the contralateral hemisphere was 0.625 for predicting postoperative CHS. The AUC for rfD* in the ACA and MCA supply area in the ipsilateral hemisphere for predicting postoperative CHS were 0.654 and 0.680.

The parameters of the IVIM sequence, which showed statistically significant differences between the CHS and non-CHS groups, were used to generate ROC curves for the prediction of postoperative CHS. The results showed that the areas under the curve (AUCs) of rf in the ACA, MCA, and PCA supply areas of the ipsilateral hemisphere for predicting postoperative CHS were 0.700, 0.733, and 0.701, respectively. The AUC for rf in the ACA supply area in the contralateral hemisphere was 0.625 for predicting postoperative CHS. The AUC for rfD* in the ACA and MCA supply area in the ipsilateral hemisphere for predicting postoperative CHS were 0.654 and 0.680. The threshold, sensitivity, and specificity of each diagnostic indicator are shown in Table 5 and Figure 3.

**TABLE 5:** Sensitivity and specificity of diagnostic thresholds for different diagnostic indicators.

	Threshold value	Sensitivity	Specificity
**Ipsilateral**			
rf-ACA	1.1548	0.704	0.639
rf-MCA	1.3787	0.778	0.701
rf-PCA	1.2059	0.667	0.629
rfD*-ACA	1.223	0.630	0.598
rfD*-MCA	1.558	0.630	0.732
**Contralatera**l			
rf-ACA	1.0951	0.630	0.505

*ACA, anterior cerebral artery supply area; MCA, middle cerebral artery supply area; PCA, posterior cerebral artery supply area.*

## Discussion

Our study used the IVIM technique to compare the microvascular perfusion status between patients with MMD and healthy volunteers and subsequently compared the preoperative microvascular perfusion status between CHS and non-CHS groups with MMD. The results indicated that the rf value was higher in the healthy control group than in the MMD group. Preoperative non-invasive IVIM-MRI analysis, particularly the *f*-value of the ipsilateral hemisphere, may be helpful in predicting CHS in patients with MMD after surgery. Patients with MMD with Matsushima types I–V are more likely to develop CHS after surgery.

In the IVIM sequence, the number of *b*-values and specific values are also related to the accuracy of the IVIM measurement parameters. In our study, referring to previous study results, a total of 16 *b*-values were set in the IVIM sequence (Federau et al., 2012). The *b*-values were 0, 20, 40, 80, 110, 140, 170, 200, 300, 400, 500, 600, 700, 800, 900, and 1,000 s/mm^2^, among which there were eight *b*-values lower than 200 s/mm^2^. The base for separate perfusion and diffusion parameters in IVIM is the signal-to-noise ratio (SNR). Wu et al. (2015) suggested that when *b* = 1,000 s/mm^2^, the minimum SNR is 30 in the brain and can obtain a reliable *f*-value to measure cerebral blood volume (CBV). In this study, the maximum *b*-value was 1,000 s/mm^2^.

D is the diffusion coefficient of free water, and ADC is the sum of the contributions of all diffusion coefficients related to the resulting motion (Le Bihan et al., 1986). Although D and ADC are different in mathematical theory, they have a strong correlation. The *D*-value, along with the ADC, is widely used in the diagnosis of acute stroke and has shown reliable diagnostic efficacy (Suo et al., 2016). In this study, we chose subjects without acute cerebral infarction to exclude different blood perfusion statuses that may influence the D and ADC values. The results showed no significant difference in rD and rADC values between the MMD patient and healthy control groups (*P* > 0.05). The IVIM microvascular perfusion parameters comprise a perfusion fraction (f), which describes the fraction of incoherent signal that arises from the vascular compartment, and a D*, which macroscopically describes the incoherent blood movement in the microvasculature compartment. A previous study showed that f represents the ratio of capillary volume in the voxel to the entire tissue volume, which can reflect the proportion of water molecules in the capillary network (Lee et al., 2014). In other words, f is related to the microvascular perfusion status of the tissues and is related to the capillary density score (Lee et al., 2014). Previous study showed that f is related to CBV, D* is related to 1/MTT, and fD* is related to CBF (Paschoal et al., 2018). In our study, the results showed that only the rf in the healthy control group was significantly higher than that in the MMD patient group (*P* < 0.05). This finding is also consistent with the hemodynamic change in MMD, which is characterized by varying degrees of steno-occlusion of the distal internal carotid artery and reduction of cerebral microvascular perfusion. The D* value is also related to the microvascular perfusion of the capillary network, although our study showed no statistically significant differences in rD* between the MMD and healthy control groups (*P* > 0.05). This result may be related to the stability of the D* value. A previous study showed that the repeatability of the D* value is poor, which may be due to the high sensitivity of this value; the D* value may be influenced by the partial volume effect caused by capillary blood flow and lacunae, which contain the cerebrospinal fluid (CSF) (Bisdas et al., 2013). In this study, patients with MMD with bilateral vascular involvement were enrolled; therefore, we chose a relatively normal ipsilateral cerebellar hemisphere rather than the other hemispheres to standardize the D, D*, ADC, and f values. The value of ADC, f, D, and D* value in cerebellum between MMD and healthy control groups were also compared, in order to evaluate the influence of crossed cerebellar diaschisis (CCD). The result showed that no significant differences were found of ADC, f, D, and D* value in cerebellum between the MMD and healthy control groups (*P* > 0.05, Table 2). However, MMD may also affect the PCAs, which may also cause some deviation in the measurements.

In patients with MMD, chronic ischemia leads to the development of new pathological vessels, with impaired cerebrovascular autonomic regulation and cerebrovascular reactivity, and the vessels are unable to control the increased cerebral blood flow after surgery. Therefore, CHS is considered to occur after surgery (Hayashi et al., 2012). The incidence of CHS in our study was 21.77% (27/124), which is consistent with a previous study result ranging from 6.7 to 38.2% (Fujimura et al., 2011, 2012). Although patient age, sex, hypertension, diabetes, and Suzuki stage showed no significant differences between the CHS and non-CHS groups in this study, the Matsushima type had a statistically significant difference between the two groups. A previous study demonstrated that adult patients with MMD commonly present with hemorrhage due to intimal tearing and subsequent blood vessel rupture (Dusick et al., 2011; Okada et al., 2012). Our study showed that approximately 34.68% (43/124 operated hemisphere) of the operative patients presented with Matsushima type VI (hemorrhage) symptoms, although among operative patients with postoperative CHS, approximately 88.89% (24/27 operated hemisphere) presented with Matsushima types I–V (ischemic) symptoms and 11.11% (3/27) presented with Matsushima type VI (hemorrhage) symptoms. The results were consistent with those of a previous study, which concluded that symptomatic hyperperfusion occurred more often in patients with ischemic onset than in those with hemorrhage (Hayashi et al., 2010).

Previous studies mainly used perfusion imaging to evaluate postoperative CHS and demonstrated that either an increase of more than 100% or a comparable postoperative increase in the regional cerebral blood flow after revascularization in patients with MMD may indicate CHS (Kaku et al., 2012; Fujimura et al., 2015). However, these radiologic techniques can only measure hemodynamics in a specific ROI. In this study, we measured the microvascular perfusion of both ipsilateral and contralateral surgery hemispheres according to different cerebral artery supply areas.

A previous study showed that although IVIM perfusion might be affected by physiological factors, such as the cardiac cycle and non-vascular components, there was a significant correlation between f and CBV, fD*, and CBF (Federau et al., 2014; Wu et al., 2015). Our study showed that in the CHS group, the rf values of the ACA, MCA, and PCA supply areas in the ipsilateral surgery hemisphere were significantly higher than those in the non-CHS group (*P* < 0.05). As mentioned above, f is related to the microvascular perfusion status of the tissues and is related to the capillary density score (Lee et al., 2014). Higher *f*-value may indicate a higher proportion of water molecules in the capillary network and may also indicate the higher density of micro collateral vessels. Consequently, areas with a higher density of micro collateral vessels may receive more blood volume after revascularization surgery. Our study also showed that the rfD* values of the ACA and MCA supply areas of the ipsilateral hemisphere were significantly higher in the CHS group than in the non-CHS group (*P* < 0.05). This may also reflect that in the CHS group, the preoperative CBF of ipsilateral hemisphere was higher than non-CHS group. Studies have shown that if revascularization surgery is performed when the cerebrovascular reserve is normal, surgery may increase the risk of postoperative CHS and may also affect the establishment of collateral circulation (Han et al., 2011). The results of this study are also consistent with those of previous studies; patients with MMD with higher preoperative *f*-values are more likely to develop postoperative CHS than patients with lower *f*-values. This finding also indicates that patients with MMD who develop postoperative CHS have better preoperative cerebral microvascular perfusion status than patients without postoperative CHS. The *f*-value for the ACA supply area in the contralateral hemisphere was significantly higher in the CHS group than in the non-CHS group (*P* < 0.05). The rf values of the MCA and PCA supply areas were not significantly different between the CHS and non-CHS groups (*P* > 0.05). We hypothesized that it might be related to the anterior communicating artery, which connects the bilateral ACA supply cerebral area. Therefore, the *f*-values of the MCA and PCA supply areas of the contralateral hemisphere were not significantly correlated with postoperative CHS.

In this study, the rADC, rD, and rD* values of all areas in both ipsilateral and contralateral hemispheres showed no significant difference between the CHS and non-CHS groups (*P* > 0.05). As mentioned above, the D and ADC values were not associated with microvascular perfusion. Each operative patient with MMD in both the CHS and non-CHS groups showed no acute infraction; thus, the D and ADC values had no significant difference between the two groups. Previous studies have shown that the D* value has poor stability and may be affected by the CSF (Wirestam et al., 2001). Intracranial ischemia and encephalomalacia are common in patients with no acute infraction. Although we tried our best to avoid cerebral sulci, intracerebral ischemia, and encephalomalacia when placing the ROIs, the CSF may affect the D* value more or less. This may explain why the D* value was not significantly different between the CHS and non-CHS groups.

We used the rf value of the ACA supply area in the ipsilateral and contralateral hemispheres to generate ROC curves for the prediction of postoperative cerebral hyperperfusion. The results indicated that the AUC for the rf values was higher than that for the rfD* values to predict postoperative CHS. And the AUC for the rf values of the ACA, MCA, and PCA supply areas in the ipsilateral hemisphere was higher than that for the rf values of the ACA supply area in the contralateral hemisphere to predict postoperative CHS. These results also indicated that the microvascular perfusion status of the ipsilateral hemisphere was more related to the postoperative CHS than that of the contralateral hemisphere. The blood flow of the anastomosed vessel increases immediately after revascularization surgery, which may directly affect the microvascular perfusion status of the ipsilateral surgery hemisphere. The microvascular perfusion status of the ACA supply area in the contralateral hemisphere may also be affected by the anterior communicating artery. With respect to the prediction of postoperative CHS, in the ipsilateral hemisphere, the AUC for the rf values of the MCA supply areas was higher than that for the rf values of the ACA and PCA supply areas. This was also consistent with the operation. In this study, all patients underwent STA-MCA bypass surgery; hence, the preoperative microvascular perfusion status of the MCA supply area may show the highest relation to postoperative CHS.

This study has some limitations. First, the sample size was small; therefore, identifying the independent predictors of CHS using multivariate logistic regression is not feasible. Second, CHS was diagnosed mainly based on the patient’s clinical symptoms, instead of the standard diagnostic method (van Mook et al., 2005; Kawamata et al., 2011). Third, this study lacked microvascular perfusion information of patients with MMD who developed postoperative CHS because most patients who developed postoperative CHS were not appropriate to perform the perfusion study. A previous study showed that f is assumed to be proportional to CBV and D* is assumed to be proportional to the reciprocal value of the mean transit time (Federau et al., 2014). This study lacked other perfusion techniques for comparison. This will be improved in future studies.

IVIM can be used to evaluate the microvascular perfusion status of patients with MMD. Preoperative non-invasive IVIM-MRI analysis, particularly the *f*-value of the ipsilateral hemisphere, may be helpful in predicting CHS in patients with MMD after surgery. Patients with MMD with ischemic onset symptoms are more likely to develop CHS after surgery.

## Data Availability Statement

The raw data supporting the conclusions of this article will be made available by the authors, without undue reservation.

## Ethics Statement

The studies involving human participants were reviewed and approved by the Ethics Committee of Huadong Hospital. The patients/participants provided their written informed consent to participate in this study.

## Author Contributions

WZ, FG, and GL contributed to conception and design of the study. WZ, FG, YZ, and ZZ organized the database. WZ and FG performed the statistical analysis and wrote the first draft of the manuscript. YZ, YD, and ZZ wrote sections of the manuscript. All authors contributed to manuscript revision, read, and approved the submitted version.

## Conflict of Interest

The authors declare that the research was conducted in the absence of any commercial or financial relationships that could be construed as a potential conflict of interest.

## Publisher’s Note

All claims expressed in this article are solely those of the authors and do not necessarily represent those of their affiliated organizations, or those of the publisher, the editors and the reviewers. Any product that may be evaluated in this article, or claim that may be made by its manufacturer, is not guaranteed or endorsed by the publisher.
