# Supplementary material for: Intravoxel Incoherent Motion Magnetic Resonance Imaging Used in Preoperative Screening of High-Risk Patients With Moyamoya Disease Who May Develop Postoperative Cerebral Hyperperfusion Syndrome
Source: Front Neurosci. 2022 Mar 2;16:826021. doi: 10.3389/fnins.2022.826021 (PMC8924456; doi:10.3389/fnins.2022.826021)
Supplement: Supplementary file 1 [file Table_1.DOCX]

Supplement Table: Matsushima symptom classification and Suzuki angiographic stage

|  | Description |
| --- | --- |
| **Matsushima type** |  |
| Type I | Transient ischemic attacks (TIAs) or episodes of reversible ischemic neurological deficit (RIND) occurring less than twice a month, and with a normal CT appearance. |
| Type II | Episodes of TIA or RIND occurring at least twice a month, with normal CT findings and neurological examination. |
| Type III | Repeated TIAs or RINDs, with low density areas present on CT or with permanent focal neurological deficits. |
| Type IV | Onset with permanent focal neurological impairment secondary to cerebral infarction, followed by recurrent TIAs or RINDs, or rarely repeated episodes of cerebral infarction. |
| Type V | Onset with neurological deficits due to cerebral infarction, followed by repeated episodes of cerebral infarction. |
| Type VI | Onset with subarachnoid hemorrhage. |
| **Suzuki stage** |  |
| Stage 1 | Narrowing of Carotid Fork. The carotid fork is only narrowed and no other abnormalities are demonstrated in this stage. |
| Stage 2 | Initiation of the Moyamoya. The next stage of this disease, the dilatation of the intracerebral main arteries. |
| Stage 3 | Intensification of the moyamoya. In this stage, defection of the middle and anterior cerebral arteries, the moyamoya is clearly demonstrated, and changes begin to appear in the intracerebral main arteries, replaced by the moyamoya which is observed in a form of rather distinctly visualized cluster of the blood vessels. |
| Stage 4 | Minimization of the Moyamoya. In this stage, the occlusion of the internal carotid artery extends as far as the junction of the posterior communicating artery, and finally the posterior cerebral artery which has been visualized so far will disappear from an angiogram. However, even in this period, the anterior and middle cerebral arteries can be traced very dimly or in a completely different shape through the mist of the moyamoya. Besides, the moyamoya will become rough in this stage, and the constituent vessels will be thin and form a poor network. The intraorbital moyamoya will conversely increase, and the collateral from the extracranial area will also enlarge gradually. |
| Stage 5 | Reduction of the moyamoya. In this stage, the whole main arteries arising from the internal carotid artery disappear completely. The minimizing and reducing tendency of the moyamoya which has started in the previous stage will further progress, the moyamoya being poorer and limited to the syphon. Besides, the obstruction will further extend downward, showing a complete occlusion as far as C_2_ or to above C_3_ in the internal carotid artery. The collateral circulation from the external carotid artery continues to increase. |
| Stage 6 | Disappearance of the moyamoya. In the terminal stage of this disease, it seems that the cerebral circulation from the internal carotid artery is completely missing, but that the cerebral circulation is maintained only by the route of the external carotid artery or of the vertebral artery. |
